# QSPRpred: a Flexible Open-Source Quantitative Structure-Property Relationship Modelling Tool

**DOI:** 10.1186/s13321-024-00908-y

**Published:** 2024-11-14

**Authors:** Helle W. van den Maagdenberg, Martin Šícho, David Alencar Araripe, Sohvi Luukkonen, Linde Schoenmaker, Michiel Jespers, Olivier J. M. Béquignon, Marina Gorostiola González, Remco L. van den Broek, Andrius Bernatavicius, J. G. Coen van Hasselt, Piet. H. van der Graaf, Gerard J. P. van Westen

**Affiliations:** 1 Computational Drug Discovery, Leiden Academic Centre for Drug Research, Leiden University, Einsteinweg 55, Leiden, 2333 CC The Netherlands; 2 CZ-OPENSCREEN: National Infrastructure for Chemical Biology, Department of Informatics and Chemistry, Faculty of Chemical Technology, University of Chemistry and Technology Prague, Technická 5, Prague, A-4040 Czech Republic; 3 Department of Human Genetics, Leiden University Medical Center, Einthovenweg 20, Leiden, 2333ZC The Netherlands; 4 ELLIS Unit Linz and LIT AI Lab, Institute for Machine Learning, Johannes Kepler University, Altenberger Straße 69, Linz, 610101 Austria; 5 Department of Neurosurgery, Brain Tumor Center Amsterdam, Amsterdam University Medical Center, Cancer Center Amsterdam, De Boelelaan 1117, Amsterdam, 1081 HV The Netherlands; 6 Oncode Institute, Utrecht, The Netherlands; 7 Leiden Institute of Advanced Computer Science, Leiden University, Niels Bohrweg 1, Leiden, 2333 CA The Netherlands; 8 Certara UK, University Road, Canterbury Innovation Centre, Unit 43, Canterbury, Kent, CT2 7FG UK

**Keywords:** QSPR modelling, QSAR modelling, Proteochemometrics, Cheminformatics, Machine learning, Software

## Abstract

Building reliable and robust quantitative structure–property relationship (QSPR) models is a challenging task. First, the experimental data needs to be obtained, analyzed and curated. Second, the number of available methods is continuously growing and evaluating different algorithms and methodologies can be arduous. Finally, the last hurdle that researchers face is to ensure the reproducibility of their models and facilitate their transferability into practice. In this work, we introduce QSPRpred, a toolkit for analysis of bioactivity data sets and QSPR modelling, which attempts to address the aforementioned challenges. QSPRpred’s modular Python API enables users to intuitively describe different parts of a modelling workflow using a plethora of pre-implemented components, but also integrates customized implementations in a “plug-and-play” manner. QSPRpred data sets and models are directly serializable, which means they can be readily reproduced and put into operation after training as the models are saved with all required data pre-processing steps to make predictions on new compounds directly from SMILES strings. The general-purpose character of QSPRpred is also demonstrated by inclusion of support for multi-task and proteochemometric modelling. The package is extensively documented and comes with a large collection of tutorials to help new users. In this paper, we describe all of QSPRpred’s functionalities and also conduct a small benchmarking case study to illustrate how different components can be leveraged to compare a diverse set of models. QSPRpred is fully open-source and available at https://github.com/CDDLeiden/QSPRpred.

**Scientific Contribution**

QSPRpred aims to provide a complex, but comprehensive Python API to conduct all tasks encountered in QSPR modelling from data preparation and analysis to model creation and model deployment. In contrast to similar packages, QSPRpred offers a wider and more exhaustive range of capabilities and integrations with many popular packages that also go beyond QSPR modelling. A significant contribution of QSPRpred is also in its automated and highly standardized serialization scheme, which significantly improves reproducibility and transferability of models.

**Supplementary Information:**

The online version contains supplementary material available at 10.1186/s13321-024-00908-y.

## Introduction

Quantitative Structure–Property Relationship (QSPR) modelling can be described as the application of empirical methods (i.e. statistical and machine learning (ML) approaches) to find the mathematical relationship between molecular structure and a property of interest [1]. In the past decades, QSPR and mainly Quantitative Structure-Activity Relationship (QSAR) modelling methods have established themselves as key instruments in drug discovery [2–6] and beyond [1, 7]. Reliable QSPR models have the potential to reduce the need for time and resource intensive experimental screening of compounds by enabling effective compound selection in the drug development pipeline [8]. The increasing amount of experimental data available (i.e., in ChEMBL [9] and PubChem [10]) also enables the use of more advanced methods which have seen rapid development [11, 12]. Therefore, given the prevalence of QSPR modelling and its constant development, there is a need for software tools that can support researchers not only in the tasks of developing and deploying models in practice, but also in the critical assessment of new methods and validation of non-trivial computational workflows that include many preliminary steps such as collection, curation and analysis of data as well as model training, evaluation and deployment.

In the traditional sense, QSPR modelling focuses mainly on describing the relationship between the compound structure and a property of interest, but proteochemometric modelling (PCM) has emerged as an extension that also introduces the protein target information into the equation [13, 14]. A PCM approach can extrapolate similarities and differences across (super)families and is therefore promising in poly-pharmacology and off-target prediction [15], as well as a strategy for data augmentation and relevant binding residue identification [16, 17]. Although in the traditional sense, the architecture is identical to that of a single-task model, it includes bio-activity endpoints for multiple proteins, by featurizing each compound-protein combination separately [18]. Therefore, PCM has inherent applicability challenges that combine those of single-task and multi-task modelling (i.e. dataset size [19, 20], data balance [21, 22], sparsity [23], but also unique featurization requirements that need to take the proteins themselves into account [19, 20]).

No matter the underlying philosophy, both traditional and PCM QSPR models can be obtained by combining various algorithms and methodologies, which often need to be benchmarked against one another in a systematic and comprehensive manner. While there is still an ongoing debate on whether and under what conditions a meaningful comparison of QSPR methodologies is possible [24], benchmarking and systematic comparison have become an integral part of the QSPR field. Whether it is the comparison of new algorithms [18, 25, 26], molecular representations [27–29], model development strategies [20, 21], model validation [30, 31], the nature of data [32], or to provide usage guidelines [33], one common denominator of such studies is that they base their conclusions on a systematic comparison using a standardized subset of data. During this task, researchers are faced with many challenges from compiling a representative subset of data for the diverse set of tasks seen in QSPR modelling [29] to choosing the right method to obtain statistically sound results [34–36]. However, even more fundamental problems such as the dominance of median predictions [24] or combining data sources [37] can plague benchmarking results. Therefore, even with the long history of the QSPR modelling field, it is clear that benchmarking methodologies are important, but not problem-free.

Another challenge that QSPR modellers face is the reproducibility of results and model deployment. While not specific to QSPR modelling [38, 39], reproducibility is a topic widely discussed in cheminformatics and computational drug discovery [40–42] and pertains mainly to correct estimation of real-world performance data, but also the practical transition from model building and evaluation to deployment [43]. For example, the deployment phase also needs to implement crucial steps to process compound structures before prediction to ensure equivalent compound representation as during training. However, currently, there is a lack of open-source tools that would sufficiently address the reproducibility of results and the transfer of models into practice and it is often up to the modeller to provide these, which leads to a large disparity between researchers in how reproducibility and deployment of models is addressed.

Several open-source applications are available that support researchers in QSPR modelling and help in solving some of the aforementioned challenges (Table 1). A popular framework is KNIME [44], which utilizes a GUI with visual workflows and has many pre-implemented components. However, since KNIME is not dedicated specifically to QSPR modelling designing custom components in KNIME can be challenging and the integration of Python extensions in the Java-based API is not always straightforward [45]. With a focus on deep-learning-based models, DeepChem [46], was one of the pioneering Python packages for molecular modelling. It offers a wide array of different featurizers and models and a flexible and easy to understand API that is modular and extensible. However, not all DeepChem models address the aforementioned reproducibility and deployment issues out-of-the-box. For example, the offered SklearnModel class does serialize the model itself, but reproducing the preparation workflow and creating the feature matrix is left to the users themselves, which can be inconvenient or, worse, might create reproducibility and deployment problems. Extending DeepChem, AMPL [47] prioritizes automated machine learning for benchmarking and it enables users to conveniently build and validate models. However, it still lacks functionality to readily deploy and use models in practice. Uni-QSAR [48] was also recently introduced as part of the Uni-Mol [49] package. However, it is heavily based around deep learning models, which results in less extensible and intuitive API. ZairaChem [50] is another recent package, which proposes an automated cascade for training machine learning models, empowering users with little knowledge in data science to train robust ensemble-based models. However, one limitation of ZairaChem is that it currently only supports classification models and also does not enable model serialization with preparation steps included. This is addressed in a recently published package, PREFER [51], which wraps trained models fully, including data preprocessing. It implements a pipeline based on AutoSklearn [52], covering steps such as data preparation, model selection, and model evaluation. However, PREFER, offers a slightly less flexible API than the previous options and combining different feature representations and splitting methods is not possible without modifying the source code of the package itself. One more package that supports comprehensive serialization of data preprocessing steps within produced models is QSARtuna [53]. It features a modular API with a variety of pre-implemented algorithms and featurizers, as well as a focus on model explainability. However, due to its focus on hyperparameter optimization and streamlining the modelling process, the API is not as rich and extensible as in the case of some other packages such as DeepChem. For example, due to the dependence of QSARtuna on the Optuna package, implementing alternative optimization frameworks might not be as straightforward. A similar package to QSARtuna is Scikit-Mol [54], which is tightly bound to the scikit-learn package and its pipelines API. It also features preparation pipeline serialization for model deployment, but lacks some advanced features such as custom data split implementations, composite descriptors or applicability domain tools. All of the aforementioned packages also lack support for PCM modelling with QSARtuna as a notable exception with its support for simple Z-scale descriptors. Even though there exists a package with pure focus on PCM, an R tool called camb (Chemically Aware Model Builder) [55], it has not been maintained since 2017 [56]. Therefore, support for accessible and straightforward PCM modelling is still lacking among contemporary open source packages. Similarly, the inclusion of applicability domain of QSPR models is also not fully considered in any of the above packages.

In this work, we present QSPRpred, an open-source package that attempts to compile the essentials of QSPR modelling into a compact and accessible Python package, which offers some advantages over already existing packages (Table 1). As such, the package aims to address a user base with varying ranges of expertise from students to well-rounded QSPR modellers interested in developing and testing new approaches. With QSPRpred we try to provide high-level interfaces to accomplish QSPR modelling tasks in few steps, but at the same time try to encourage writing of modular Python code by making sure all variable steps are encapsulated and easily replaceable by custom implementations. In addition to being customizable, all steps can also be combined using the built-in benchmarking workflow, which enables streamlined model building to the likes of AMPL or QSARtuna, but completely described in Python with “plug-and-play” components. This provides a platform for researchers to experiment and validate novel approaches quickly. In addition, QSPRpred tackles problems related to reproducibility, transferability and deployment of models. While reproducibility is ensured by streamlining setting of random seeds, QSPRpred also provides a global serialization API that not only includes the model itself, but also the molecule preparation and featurization steps. This simplifies the deployment of models and also makes them transferable since the shared model can be reloaded and directly used for predictions from SMILES strings without the need to repeat any preparation steps.

**Table 1:** Comparison of QSPR modelling tools (adapted from Mervin et al. [53])

Property	QSPRpred	QSARtuna [53]	AMPL [47]	PREFER [51]	Uni-QSAR [48, 49]	Scikit-Mol [54]
Dataset modellability/premodelling evaluation	no	no	no	no	no	no
Custom splitting techniques	yes	yes	no	no	no	no
Number of descriptors	10+	8	4	4	5+	9
Composite descriptors	yes	yes	no	no	no	no
Custom descriptors	yes	yes	no	no	no	yes
Custom train/test splits	yes	yes	no	no	no	no
Shallow models	yes	yes	yes	yes	yes	yes
Neural network-based algorithms	yes	yes	yes	yes	yes	no
Inductive model calibration	no	yes	no	no	no	no
Uncertainty estimation	no	yes	yes	no	no	no
Explainability	no	yes	no	no	no	no
Multiparameter optimization	yes	yes	no	no	no	yes
Probabilistic transform	no	yes	no	no	no	no
Applicability domain	yes	no	no	no	no	no
Continuous integration/automatic testing	yes	yes	yes	no	no	yes
Model transferability	yes	no	yes	yes	no	yes
proteochemometrics	yes	yes	no	no	no	no

Recreation of the comparison drawn by Mervin et al. [53] with the QSPRpred framework added. The table was slightly modified to reflect features of QSPRpred that were not previously considered in the comparison (“continuous integration and automatic testing”, “model transferability”, and “proteochemometrics”)

In addition, the "Neural network-based algorithms" category for Uni-QSAR was set to "yes" because we believe it was mistakenly set to "no" in ref. [53]. The authors of Uni-QSAR even state "In addition to neural network models, some classical machine learning algorithms such as Extreme Tree, Gradient Boosting Decision Tree, Support Vector Machine, etc. are also integrated into our framework." [48]

## Implementation

**Fig. 1:**
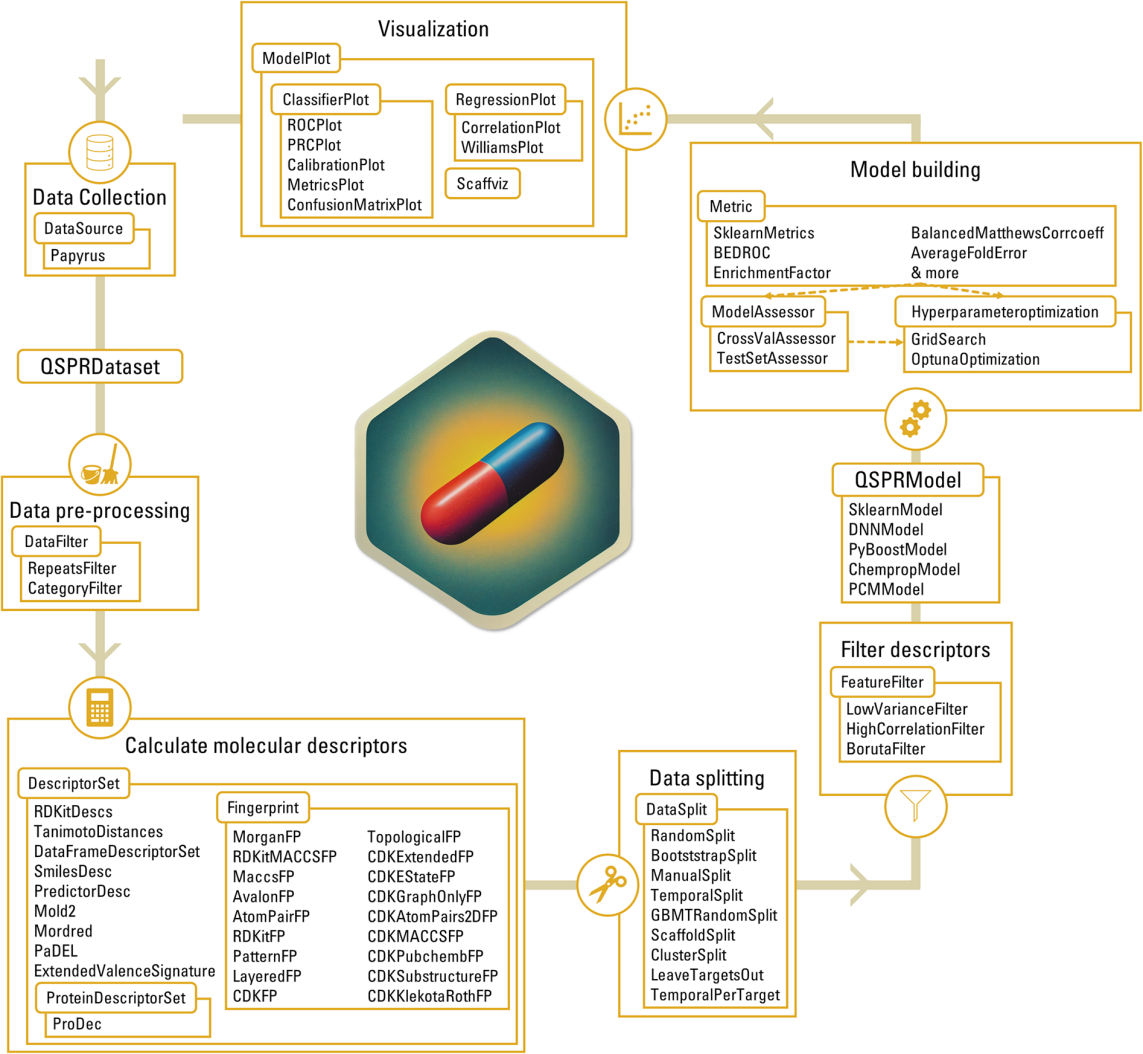
Visualization of the QSPRpred workflow. Each box represents a general step in QSPR/QSAR modelling, e.g. data collection and visualization. Every rounded rectangle is an abstract base class in QSPRpred defining the interface of the respective step. Each of these classes has a number of implementations included, which are listed in the attached box, e.g. the abstract base class DataFilter has RepeatsFilter and CategoryFilter available as an out-of-the-box implementation. Therefore, altering the behaviour of each component can be achieved either through inheritance or by simply providing a custom implementation if the respective abstract base class

Although a wide variety of different QSPR models and descriptors have been described in literature [1], there is a significant overlap in the general QSAR/QSPR modelling workflow. QSPRpred leverages this by providing a modular framework that comes with many out-of-the-box components while also providing clear interface definitions to facilitate comprehensive extensibility features (Fig. 1). The description of various aspects of this workflow will be the subject of the following subsections: Section Data includes an extensive description of all the data preparation components that QSPRpred provides. Section Modelling provides relevant details on model training and evaluation. In addition, QSPRpred also provides various visualization tools, which are described in section Visualization. The API features presented in this work are consistent with the latest QSPRpred version at the time of writing (v3.2.0).

### Data

#### Data collection

The first step of any QSPR modelling project is the collection of data. The supplied data should at least contain the molecule SMILES sequences and the property to be predicted, but can contain any extra information as needed. The standardization of SMILES strings can be handled separately by the user, but QSPRpred also provides a molecular storage and registration API that can be leveraged for that purpose (currently available in a pre-release version of the package).

Furthermore, data can be retrieved from an external source programmatically, using the DataSource class, which is used to describe the creation of data sets from different sources. By default QSPRpred provides the Papyrus data source, which can be used to collect data from the eponymous dataset Papyrus, a large-scale curated bioactivity dataset [57]. This method of data collection is also used for creating multiple benchmarking datasets dynamically, see section Experiment 1: benchmarking single-task and multi-task regression models for an example. Fetching data with a DataSource will directly return the data in a MoleculeTable object or a QSPRDataset. MoleculeTable is a data container that provides functionality to handle different operations for molecule preparation and descriptor calculation while QSPRDataset is its extension that provides functionality specific to QSPR modelling such as data splitting.

An instance of QSPRDataset can also be initialized directly (without a DataSource) by the user through a tabular format (e.g. CSV/TSV) or an SDF file. The user always needs to specify one or more properties to be predicted during initialization or during the lifetime of the data set. These are specified as Target Property, which are associated with a specific modelling TargetTask, such as REGRESSION or MULTICLASS.

In the following sections, several data pre-processing and preparation steps will be described which can be applied to the QSPRDataset. The prepareDataset method of QSPRDataset streamlines the process by applying the data preparation steps in a fixed order: data filtering, descriptor calculation, data splitting, feature filtering and feature standardization. The user can specify which components should be used for each step, which includes custom components that can be implemented by creating subclasses of the abstract base class for each component. The QSPRDataset and MoleculeTable are serializable to JSON (JavaScript Object Notation) and other associated files (see section Reproducibility & Transferability), which enable the user to save and reload prepared datasets in machine- and human-readable format.

#### Data pre-processing

Before being used for model fitting, data samples may be removed based on the following criteria: specific values (CategoryFilter, e.g. remove all samples from a specific source or year), identical descriptor values (RepeatsFilter) or user-defined filters. Moreover, as the underlying pandas [58] dataframe can be accessed and edited, any further data analysis steps can be executed in this phase. For easy visualization and exploratory analyses of the dataset, integration with Scaffviz [59] is provided, which is described in more detail in the visualization section Visualization.

#### Descriptor calculation

Descriptor calculation in QSPRpred is facilitated through the DescriptorSet class which wraps one or more types of descriptors. There are many integrated implementations of the DescriptorSet available including: RDKit descriptors [60], Mordred [61], Mold$^2$ [62], PaDEL [63] and Tanimoto distance to other molecules. Additionally a range of different types of molecular fingerprints are implemented, such as Morgan and MACCs [64] fingerprints. A trained QSPRpred model can itself also be used as descriptor for stacked modelling. While users can add new implementations of DescriptorSets this may not always be practical, for example when using descriptors from experimental measurements. In this case, the descriptors can be provided as a data frame directly via the built-in DataFrameDescriptorSet. Moreover, a custom implementation of the DescriptorSet interface, also exists for protein descriptors (ProteinDescriptorSet) that are commonly used for PCM modelling (i.e. z-scales [65], BLOSUM [66] and VHSE [67]). This is provided via the standalone ProDEC [68] package, which enables calculation of multiple sequence alignment-based descriptors for proteins. The multiple sequence alignment is done with Clustal Omega [69] or MAFFT [70], but QSPRpred also describes an API to easily integrate other alignment methods.

#### Feature filtering

Feature filtering is commonly used to select the most informative features from the calculated descriptors. Here, the feature filters currently implemented in QSPRpred will be discussed, however, new feature filters can be easily added by the user. Filters in QSPRpred are always calculated using only the training set or the training fold of the data set to avoid data leakage (see subsection 2.1.5). The first filter that is implemented is the LowVarianceFilter, which calculates the variance within a feature on the training set and removes it if it is below a threshold specified by the user. A HighCorrelationFilter is also available. It calculates the correlation between features and removes them if it is higher than the user-specified threshold. Finally, QSPRpred provides integration with BorutaPy [71], which is a Python implementation of the Boruta filter [72]. Boruta filtering is an all-relevant feature selection method that removes features based on their relevance compared to random features.

#### Data splitting

The choice of how to split data can greatly influence our impression of future performance of the model [22, 73]. QSPRpred supports any scikit-learn-style [74] data splitter class that has a method split(X,y) that yields for each split/fold the indices for each subset. Splits can be applied at two levels, during dataset preparation to create the independent test set (which will be used by TestSetAssessor) and during model optimization/training to create (cross-)validation sets with CrossValAssessor (described in section Model Assessment). QSPRpred also offers several integrated splits. All of these methods support the creation of a single train-test split or *k*-folds. Firstly, SklearnRandomSplit which wraps scikit-learn’s (Stratified)ShuffleSplit method. Three splits (RandomSplit, ClusterSplit, ScaffoldSplit) are included that utilize the BalanceSplit [75] package to create well-balanced data splits for (sparse) datasets without data leakage between different tasks. The ManualSplit can be used to apply a predefined split. The TemporalSplit is used to make time series-based test or cross-validation splits. QSPRpred also has a BootstrapSplit class that can wrap any split and use it for repeated sampling of the dataset applying the specified split in replicates. Furthermore, any of the above-mentioned splitters can be applied to PCM modelling with the help of the PCMSplit class, which will ensure the splits are balanced with respect to the protein targets. Two other PCM-specific splitters are also available LeaveTargetOut and TemporalPerTarget. LeaveTargetOut will remove all the data points for a certain target, to evaluate how well the PCM model extends to new targets. The TemporalPerTarget split applies a temporal split that avoids data leakage with multiple tests for compounds for different targets over time, by using the first occurrence of the molecule in the dataset for all proteins as timepoint.

### Modelling

#### General

QSPRpred inherently supports both single and multi-task variants of regression and single-class and multi-class classification. All models implemented with QSPRpred are wrapped in a QSPRModel subclass, which can then be applied to a QSPRDataset instance. The model task is automatically derived from the target properties specified in the dataset, i.e. two single-class TargetTask target properties, will result in a multi-task single-class model. Model tasks in QSPRpred are encoded as a Python Enum class (e.g. REGRESSION, SINGLECLASS, MULTITASK_MULTICLASS), which allows easy specification of which tasks a model can support. Like the dataset object, a QSPRModel can be serialized to JSON (see section Reproducibility & transferability). Currently, available model implementations are: SklearnModel, DNNModel, PyBoostModel and ChempropMoleculeModel. The SklearnModel is a wrapper for all scikit-learn estimators [74]. The DNNModel is a PyTorch [76] implementation of a fully-connected neural network. PyBoost is a wrapper around the Py-Boost [77] package, a Gradient Boosted Decision Tree toolkit. The ChempropMoleculeModel wraps the basic Chemprop [78] message passing neural network, although it does not support all functionality that Chemprop provides. Moreover, any new type of model can be simply implemented by creating a subclass of the abstract base class QSPRModel, requiring the user to just implement methods for fitting, predicting and serialization of the model. There is also a dedicated tutorial to implementing new models (see section Tutorials) to help QSPR practitioners with integration of novel methods.

#### PCM modelling

Creating a PCM model differs slightly from creating a standard QSPR model. PCM models require protein featurization and can introduce the need for different splitting methods, as discussed in section Descriptor calculation and Data splitting respectively. To create a PCM dataset, QSPRpred provides a class called PCMDataSet which forms the alternative input for a model of the class PCMModel, which is slightly altered from the base QSPRModel and can be used to wrap QSPRModel implementations for PCM. This is mainly necessary because in order to make predictions with a trained model, the protein identifier of the protein to make predictions for is needed.

#### Model assessment

To evaluate model performance, QSPRpred provides a class called ModelAssessor that defines a structure for performance evaluation. Given a QSPRpred model and a dataset, it will run an evaluation and return the specified metric. Pre-implemented or custom scoring functions can be provided to the ModelAssessor. Pre-implemented functions include balanced classification metrics [79] and all scikit-learn [74] scoring functions.

By default, two ModelAssessor subclasses are implemented, namely the TestSetAssessor and the CrossValAssessor. As the name suggests, the TestSetAssessor evaluates the model performance on the test set of the provided dataset. It will use the model to predict values for the test set and return the score given by the provided metric. On the other hand, the CrossValAssessor can perform cross-validation on the training set. The folds are determined by the user-specified splitting method (see section Data splitting). It will return a list of scores for each fold. Because of the flexible splitting method, this class is not limited to cross-validation, but can also perform bootstrapping, through resampling of the training set (see section Data splitting).

Both described ModelAssessor implementations will write the predictions to the model directory in TSV format, including unique molecule identifiers provided by the dataset. Using the identifiers each prediction can be linked back to the original data point, which allows for detailed analysis and visualization (see section Visualization) of the model performance.

#### Hyperparameter optimization

Finding the right hyperparameters for a model, can be a challenging task. As with previously discussed components, QSPRpred provides a flexible base class HyperparameterOptimization. Hyperparameter optimization requires specification of the search space; which model parameters to tune and for which values or within which bounds. A template of how to provide the search space file can be found in the documentation [80]. Furthermore, a ModelAssessor needs to be specified, that determines how a set of hyperparameters will be evaluated. If the ModelAssessor returns multiple scores, e.g. in the case of cross-validation a score for each fold is returned, the score will be aggregated by a user-specified function.

Two default implementations of HyperparameterOptimization exist: GridSearch and OptunaOptimization. GridSearch is an exhaustive search algorithm, that evaluates all combinations of the specified hyperparameters. OptunaOptimization is a form of Bayesian optimization using Optuna’s [81] Tree-structured Parzen Estimator algorithm as the acquisition function. Bayesian optimization is used for iteratively proposing new hyperparameters for a machine learning model applying the Bayesian principle, which allows for finding the optimal hyperparameter combinations without an exhaustive search. The user needs to set the number of iterations because it is an iterative process. By default the search starts with a random sample of ten parameter combinations, afterwards, the acquisition function is used.

#### Applicability domain

For evaluation of the applicability domain of trained models, QSPRpred provides integration with MLChemAD [82]. MLChemAD implements many commonly used definitions of the applicability domain for cheminformatics models, based on k-nearest neighbors, the local outlier factor or on bounding approaches. It is also possible to implement a custom applicability domain using the ApplicabilityDomain base class. An ApplicabilityDomain object may be attached to a QSPRDataset. Then during the dataset preparation, the applicability domain can be fit on the training set to identify or remove outliers from the test set. Furthermore, the ApplicabilityDomain object can be attached to a model and fit on the whole dataset. In production mode, when predicting from SMILES the model will return whether this compound is within the applicability domain of the trained model.

#### Visualization

The test set and cross-validation assessments write to result files in the model directory as described in section Model Assessment. These result files are human readable and can be used to easily generate any visualization that users require. QSPRpred also has a ModelPlot class, that provides a number of different plots that can be generated directly from an instantiated model with result files present. These plots include receiver operating characteristic (ROC) curves, precision-recall curves, calibration plots and barplots for a range of scikit-learn [74] classification metrics. Metrics such as precision, recall and Matthews Correlation Coefficient can be visualized not only for single-task models, but also for multi-task and multi-class classification models. In the case of multi-class models metrics are calculated per class (one-vs-rest) and with different averages. Moreover, correlation plots can be generated for multi and single-task regression models. In addition to the native ModelPlot, QSPRpred’s MoleculeTable and QSPRModel instances can be used directly with the interactive chemoinformatics visualization package Scaffviz [59]. Scaffviz offers alternative visualization of model errors and is essentially an adapter between molplotly [83] and QSPRpred. It can be used to apply dimensionality reduction methods to obtain 2D embeddings of molecules from descriptors and display them in an interactive scatter plot. However, any properties from the data set can be displayed on each axis as well. Points may be coloured by any property in the data set, including training and test splits. It can also visualize model errors for mispredicted compounds as color overlay as well, which helps identifying difficult compounds to predict.

#### Reproducibility & transferability

In accordance with the R(eusability) of the FAIR principles [84], almost everything in the QSPRpred API is serializable to a human-readable file format. The vast majority of objects are serializable to a JSON file, which can be read easily even without QSPRpred, including model parameters of created models and workflow settings. This is possible thanks to the ml2json package [85] and the jsonpickle [86] project. Since pandas.DataFrame instances are used to represent all tabular data, they can be saved to several formats, including human-readable .csv files. When a human-readable representation is not possible (i.e. with deep learning models), sufficient metadata is saved to be able to recreate it as closely as possible. Additionally, the random state of QSPRpred is globally configurable, allowing for full reproducibility of results involving random operations. The random seed, whether newly generated or passed by the user, is saved to metadata and can be re-used to get the same results. Each dataset is initialized with a single random state, which is adopted by models and used in all subsequent random operations. However, QSPRpred also allows further fine-grained control over the random state by having the option to control the random state of models and splits separately from the dataset.

### Architecture

**Fig. 2:**
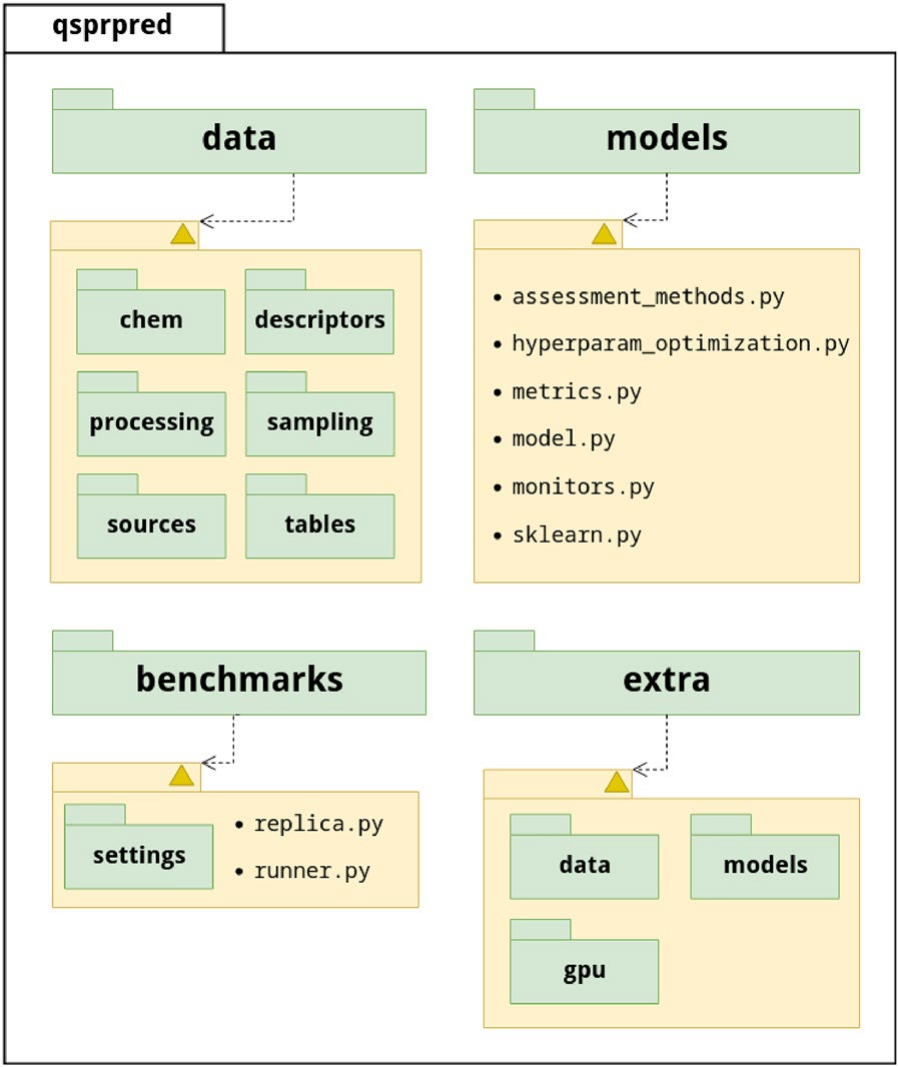
Graphical overview of the QSPRpred package architecture. Only the main packages discussed in this work are included. The majority of the functionality is centered around the qsprpred.data package, which integrates API definitions and tools to work with chemical structures (qsprpred.data.chem), featurization (qsprpred.data.descriptors), processing tools for data and feature filtering qsprpred.data.processing, data splitting and resampling (qsprpred.data.sampling), data source adapters (qsprpred.data.sources) and data storage implementations (qsprpred.data.tables). The qsprpred.models package contains the base definition of a QSPRpred model API in qsprpred.models.model, which is implemented for scikit-learn models in qsprpred.models.sklearn. In addition, the qsprpred.models package also contains functionality needed to monitor training (qsprpred.models.monitors), optimize (qsprpred.models.hyperparam_optmization) and assess model performance (qsprpred.models.assessment_methods and qsprpred.models.metrics). The qsprpred.extra package extends functionality of both qsprpred.data and qsprpred.models with additional extensions that support deep neural networks and PCM modelling functionality. Finally, the qsprpred.benchmarks package houses data classes needed to set up (qsprpred.benchmarks.settings) and run benchmarking experiments (qsprpred.benchmarks.runner)

The structure of the QSPRpred package reflects the intended usage as outlined in Fig. 1 with several packages and subpackages separating the different tasks (Fig. 2). Every data structure and functional element of the API has its own abstract definition, which is followed in the reference implementations. For example, the main QSPRDataset (located in qsprpred.data.tables.qspr) class implements several interfaces defined in qsprpred.data.tables.base. Likewise, the QSPRModel abstract base class (located in qsprpred.models.base) defines the API of a model while the qsprpred.models.sklearn is its implementation that facilitates a compatibility layer between the scikit-learn [74] package and QSPRpred. Such a standardized approach to API development makes integration of new tools and data structures easier and changes to code minimal as libraries update over time.

The modular architecture of QSPRpred also makes optional installation of dependencies possible. Much of the functionality located in qsrppred.extra depends on external packages that can sometimes pull many dependencies alongside them, but with careful modular separation it is not necessary to install those dependencies unless they are truly needed. Therefore, QSPRpred also supports various installation flags to make sure only necessary dependencies are installed for different intended use cases. For example, the dependencies needed to support functionalities in qsprpred.extra.gpu are only installed if the [gpu] flag is specified upon installation, but without it the rest of the package will still function normally.

In addition to the flexible Python API, QSPRpred also offers extensive command line interface (CLI). While less customizable than the API, the CLI allows the user to train a wide variety of QSPR models without having to write any code. The QSPRpred CLI is subdivided into three callable scripts (data_CLI, model_CLI and predict_CLI) that cover the entire QSPR model building workflow (see Fig. 1).

With the data_CLI it is possible to create a range of datasets for different tasks with one command, including datasets for multi-class and multi-task modelling (see section Modelling). Furthermore, most of the base QSPRpred data pre-preprocessing functionality is available through this CLI, including all the different descriptors sets, and data splits as well as target imputation for multi-task modelling.

After creating one or more datasets through the CLI or the Python API, the model CLI can be used for model training. With the model CLI a range of scikit-learn [74] models and a PyTorch [76] fully-connected neural net can be trained. The model_CLI provides the same main training steps as the Python API, namely hyperparameter optimization, cross-validation and test set predictions. Finally, after model training has completed, the trained models can be used to make predictions on new sets of molecules with the predict_CLI.

### Documentation

In QSPRpred, much effort is also devoted to guiding new users as well as potential contributors. The code follows a predetermined style guide [87], which requires that every functionality needs to be properly documented via a docstring that is then visible on the QSPRpred documentation page [80] upon publishing a new release. The style guide also requires that Python type hints are present for all methods and functions to indicate which data structures are compatible and expected. Pre-commit hooks are also available that can be used to check code before committing to ensure compliance with the style guide. In addition to these strict API documentation requirements, the documentation pages also contain guides on how to use the CLI and other miscellaneous items pertaining to the usage of the package.

### Tutorials

We have dedicated several Jupyter notebooks to featuring real-life QSPR modelling examples. The tutorials expect basic understanding of QSPR modelling concepts and are designed to mainly showcase QSPRpred functionality. While they are easily accessible by beginners and are suitable to be used by students, they also include specialized notebooks for more advanced users who want to customize behaviour and integrate new methods.

The user progressively learns all functionalities within QSPRpred from data set acquisition to model evaluation. A quick start tutorial is designed to get the user to prepare a dataset and run a single-task QSAR regression model with QSPRpred as quickly as possible. After the quick start tutorial, a series of one, seven and three tutorials covering respectively the basics of benchmarking, data handling and modelling within QSPRpred are available. These tutorials go over the main aspects that need to be taken into account in any modelling project prior to modelling (i.e data collection, preparation, featurization and splitting), but also the necessary tools to build and validate models (i.e. formulation of model tasks, model assessment and logging of progress and results). These basic tutorials can be accessed individually or followed sequentially from the quick start guide.

On top of the basic tutorials, a series of ten advanced data and modelling tutorials are available. These help the user to build on top of the already acquired basic knowledge of QSPRpred by teaching them how to customize functionalities and add new features. These tutorials are aimed at researchers who develop new methods and want to take advantage of QSPRpred to automate and standardize certain tasks. The advanced tutorials showcase the possibilities to perform hyperparameter optimization and add custom descriptors and data splitting methods, as well as custom models. Moreover, these tutorials also dive deeper into the modelling options by showing how to build deep learning, multi-task, and PCM models, and advanced model monitoring via the popular Weights & Biases (W &B) framework [88].

### Testing

In order to ensure that all functionalities of the package remain operational even as the code changes, be it by internal factors (code updates) or external (dependency updates), frequent testing of the code is necessary. Therefore, the inherent part of the QSPRpred package is unit testing and every new functionality added needs to be accompanied with testing code.

QSPRpred also takes advantage of continuous integration (CI) not only to run unit tests, but also to frequently run the tutorial code and check consistency of models. Therefore, it is always ensured that upon any modification of the code all tutorials are up to date, all code is working as expected and all reference models return the same expected results. The latter is especially important to guarantee model consistency across different versions of the code and its dependencies by highlighting changes that could lead to past results being unrepeatable.

## Results

QSPRpred also provides an overarching API to conduct benchmarking experiments. These features are located in the qsprpred.benchmarking package and provide a streamlined way to test various combinations of molecular descriptors, model algorithms and even data set preparation strategies. In this section, we will show two example scenarios of experiments focused on building and comparing regression models. The code to reproduce these experiments is available at https://github.com/CDDLeiden/qsp-bench, but the repository can be easily extended and adapted to other scenarios as well using the instructions within.

### Experiment 1: Benchmarking single-task and multi-task regression models

A multi-task modelling approach can be beneficial in modelling several endpoints at once for similar biological targets [89]. However, it is not always clear if such an approach will lead to an improvement over the more traditional single-task modelling or what multi-task methodology would be the most optimal for the data at hand. There is a plethora of methods that could be considered. As a result, researchers are often confronted with a large selection of viable workflows and methods [90].

For this small case study, we chose a bioactivity data set of 4 adenosine receptors (A1, A2A, A2B and A3). The adenosine receptors are a highly conserved family of receptors that share many similarities and selective modulators of adenosine receptors are of interest in drug discovery. Therefore, many compounds that share structural similarities are often tested against multiple or all of these receptors. This makes multi-task modelling an eligible method to consider when creating a QSAR model for these receptors. The data set used in this study was assembled by querying the Papyrus data set (version 05.6) on the respective UniProt accession keys (P30542, P29274, P29275 and P0DMS8) using QSPRpred’s integration. Only minor modifications to the adapter were made through inheritance to facilitate multi-task modelling and the adapted implementation is available in the qsp-bench repository. Compounds in this data set were represented by Morgan fingerprints with radius 3 and bit length of 2048 (as implemented in RDKit [60]).

The choice of models, in this case, study was motivated by the recently added multi-task capability to the popular xgboost package [91]. Since the models implemented in this package adhere to the scikit-learn API, they can be readily used in QSPRpred with the SklearnModel class. For the multi-task scenario, we compared the two modelling strategies currently implemented by xgboost: (1) Multi Output Tree (MOT) and (2) One Output Per Tree (OOT).

The goal of the case study was to compare these two multi-task strategies with a baseline KNeighborsRegressor algorithm from the popular scikit-learn package [74], but also against a simple MedianDistributionAlgorithm model inspired by the recent work of Janela et al. [24], which predicts the median target property value for every test instance. In all workflows, the target property was the median pCHEMBL value as determined from the Papyrus data set [57]. All experiments were conducted in 30 replicas and using either a standard random train-test splitting strategy or a cluster split strategy, RandomSplit and ClusterSplit classes in the QSPRpred API, respectively. For each model, we report the $R^2$ metric on each task separately 3.

**Fig. 3:**
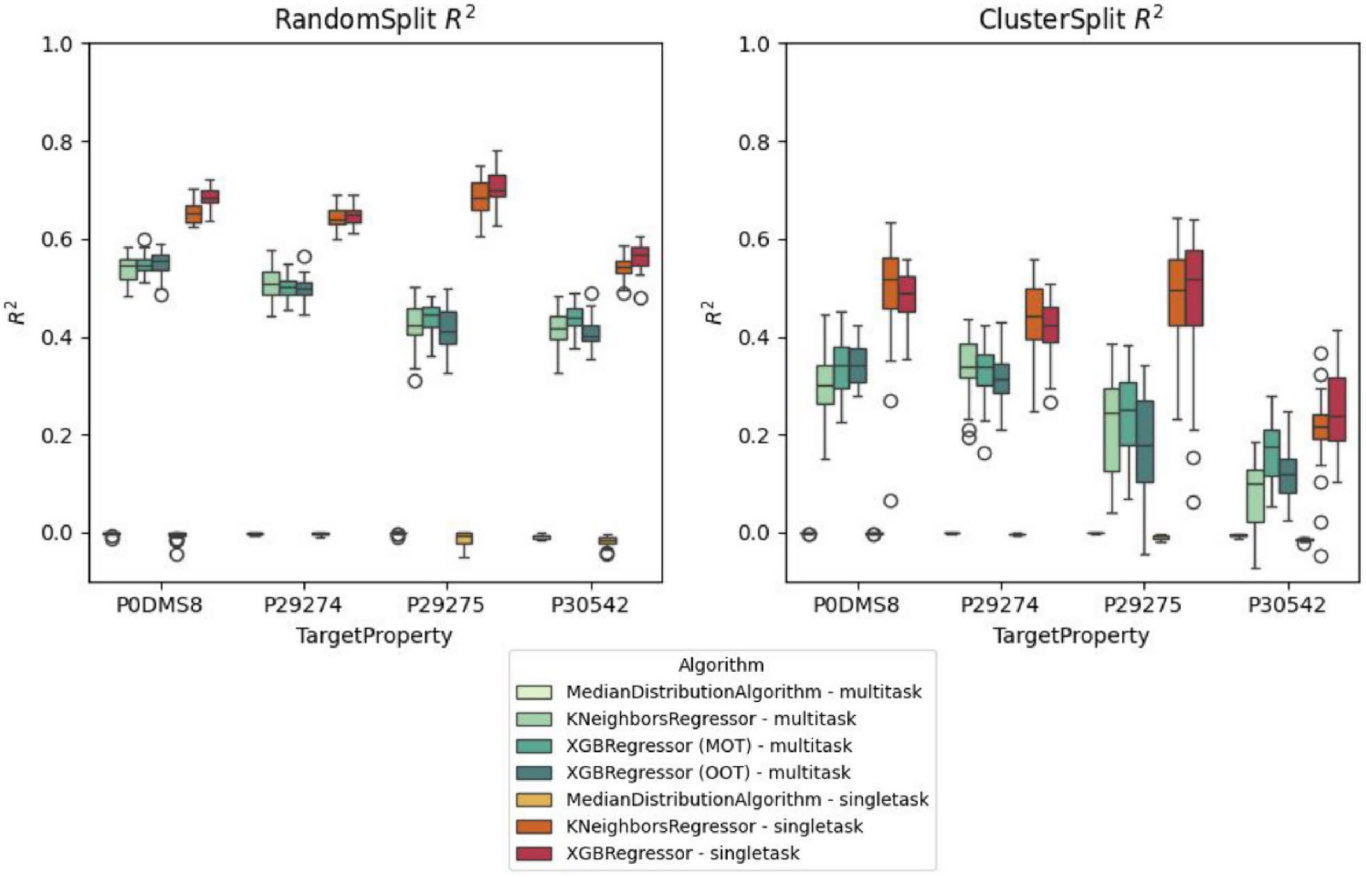
Coefficients of determination ($R^2$) calculated for each replica in different benchmarking runs conducted in Experiment 1

Overall, we found no benefit in using a multi-task model over a single-task model when using 30 repeated experiments (Fig. 3). In fact, the multi-task models showed worse performance overall in both the random split and clustered split in all tasks (Fig. 3). This is likely due to the sparsity of the multi-task target variables and the effect of imputation.

It can also be seen that the cluster split is more difficult than a standard random split for all models as indicated by a significant drop in performance for both single-task and multi-task models (Fig. 3). This is expected since the clustered split is designed with the intent to present the model with more difficult examples in the test set.

In addition, the performance shows more variance across the scaffold split than the random split. This is likely due to the difficulty of the test set fluctuating more with the varying similarity of the test compounds to the training set, which depends on the clusters created (Fig. 3).

In all experiments the xgboost models were comparable to the KNeighborsRegressor model (Fig. 3). For the multi-task case the OOT strategy worked slightly worse or was comparable to MOT (Fig. 3). Contrary to findings of Janela et al. [24], we did not see elevated performance of the MedianDistributionAlgorithm baseline on this particular data set and benchmarking conditions.

Therefore, using multiple single task models is likely a better choice for the data at hand. However, it should be noted that more models and strategies could be considered here and also compared in a follow up study. For example, deep learning models may be better-suited for multi-task modelling than the algorithms tested herein. Their architecture can be adapted in a number of ways to accommodate multi-task learning [92]. Another point to consider is the influence of sparsity of the data [93]. We simply imputed missing labels with a median value in our experiments, which may have introduced large bias to these central values in the multi-task models presented and it also affected the calculation of the final metrics. For example, when looking at RMSE an inverse pattern emerges since this metric is not sufficiently robust in this case (see Supplementary Figure S1). Therefore, proper care should be taken when evaluating models based on imputed target properties. Finally, it should also be noted that proper statistical testing should also be conducted to determine under which conditions the performance of models truly significantly differs. This was out of scope for the current work, but it would be a possibility with the obtained data.

### Experiment 2: Comparison of regression models of different architectures

One problem that QSPRpred is trying to address is how to bring models built with different algorithms and, thus, different software requirements under one roof and how to run and benchmark them with as similar API as possible. Therefore, in this case study we show an example that integrates and compares both the XGBoostRegressor model and a deep learning based method ChempropMoleculeModel in one benchmarking experiment on several data sets. Both models have different hardware and software requirements with XGBRegressor being CPU-based and accepting fingerprints as input and ChempropMoleculeModel requiring GPU-accelerated training and raw SMILES as input. With the exception of raw standardized SMILES for ChempropMoleculeModel, the same fingerprint, replica counts, splitting strategies and evaluation metrics were used in this case as well. The MedianDistributionAlgorithm was again used as a simple baseline.

**Fig. 4:**
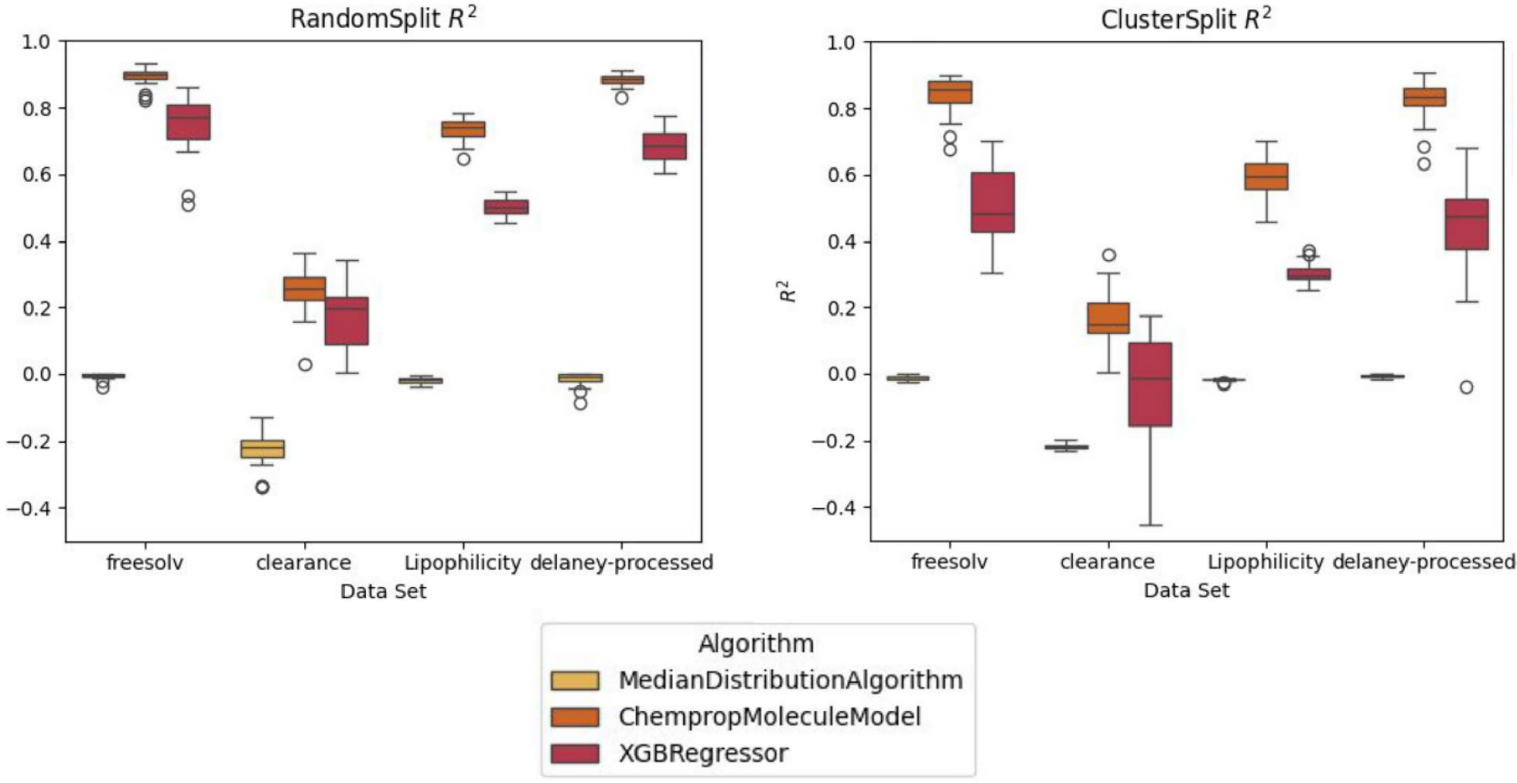
Coefficients of determination ($R^2$) calculated for each replica in different benchmarking runs conducted in Experiment 2

We also switched to four MoleculeNet data sets for this experiment to show how data sets from this source can be integrated for benchmarking. The data sets we chose have a diverse set of target properties. In particular we chose to evaluate the predictivity of the built models on the lipophilicity, clearance, solubility and free solvation energy (Fig. 4).

Clearance is known to be a difficult property to predict [94] and was also the worst performing data set in our experiments (Fig. 4). In the cluster split scenario, the XGBRegressor did not even perform above the MedianDistributionAlgorithm baseline (Fig. 4). The XGBRegressor was also the inferior model in all scenarios we observed. However, it should be noted we did not optimize hyperparameters for these models and just used them with their defaults. Therefore, it is possible a better performance could still be achieved for XGBRegressor as well as ChempropMoleculeModel. Again, we observed that the clustered split showed degraded performance across all data sets and the variance between replicas was larger (Fig. 4). This is consistent with Experiment 1. The XGBRegressor also showed larger variance in predictions as compared to ChempropMoleculeModel, which might indicate that ChempropMoleculeModel is more stable and consistent in its predictive performance.

These case studies were not by all means complete or exhaustive, but they illustrate how QSPRpred could be used to run various experiments for validation of novel QSPR methodologies on a variety of problems. Moreover, the available code in the qsp-bench repository can be quickly derived from to create other benchmarks. We think that this is an especially interesting prospect for developers of new models who can validate their approach within QSPRpred, but the simple act of integrating it into the benchmarking workflow also makes it readily available for deployment by others.

## Conclusions

QSPRpred is a new and versatile open-source package for QSPR modelling. QSPRpred addresses a number of issues in the QSPR modelling field, including a need for tools that facilitate easy comparison and validation of an ever-growing number of different QSPR workflows. QSPRpred enables this by its modular Python API that simplifies the implementation of a plethora of QSPR modelling tasks, which can be easily tied together in its benchmarking workflow. Furthermore, reproducibility of results is ensured through consistent serialisation of models and data in human-readable format. Inclusion of data pre-processing and featurization steps with the models enables direct application of trained models to new compounds using only a SMILES string. Moreover, to our knowledge this is the first QSPR modelling tool to support proteochemometric modelling in Python. QSPRpred is also integrated with a number of other packages developed in the Leiden Computational Drug Discovery group, notably DrugEx [95] for *de novo* drug design and Papyrus [57] for collection of bio-activity data. Extensive documentation and comprehensive tutorials are available.

In the future, we will continue to develop QSPRpred and extend its capabilities (Table 1). Most notably we intend to further extend the range of available descriptors (i.e. Molfeat from datamol.io [96]) and models (i.e. by adding support for ensemble modelling and a wider range of neural network architectures with the help of skorch [97], a scikit-learn [74] compatible neural network library that wraps PyTorch [76]). We will expand the applicability domain and model implementations with uncertainty estimation through conformal prediction integration [98]. Moreover, we will enrich the API with improved model calibration and explainability features. Furthermore, we will integrate QSPRpred within GenUI [99], which provides an accessible user interface for cheminformatics, QSAR modelling and AI-based molecular generation provided by the associated DrugEx framework [95]. Furthermore, continued efforts are needed to teach and adhere to high standards for FAIR [84] research practices and we hope that QSPRpred will prove to be a helpful tool to assist researchers in reproducible and transferable QSPR modelling. Therefore, with an assortment of supporting and depending packages already developed or under active development and with active user base from both Leiden University and UCT Prague, we aim to keep QSPRpred up to date and help researchers to perform experiments faster and in a more standardized and reproducible manner as the field of QSPR modelling evolves.

**Availability and requirements**

- **Project name:** QSPRpred
- **Project home page:** https://github.com/CDDLeiden/QSPRpred
- **Case Study Code:** https://github.com/CDDLeiden/qsp-bench
- **Operating system(s)** Full support for Linux. QSPRpred is supported on Windows apart from the PCM modelling, which relies on Clustal Omega [69] or MAFFT [70]. These require manually installation on Windows. We are currently working on full support for MacOS.
- **Programming Language**: Python
- **Other requirements**: Python 3.10, see https://github.com/CDDLeiden/QSPRpred/blob/main/pyproject.toml for full list of requirements.
- **License:** MIT

## Supplementary Information

**Additional file 1.**

## Data Availability

The version of the package described in this work (v3.2.0) is available on Zenodo (https://zenodo.org/records/13538072) or GitHub (https://github.com/CDDLeiden/QSPRpred/releases/tag/v3.2.0).
