# Supplementary material for: QSPRpred: a Flexible Open-Source Quantitative Structure-Property Relationship Modelling Tool
Source: J Cheminform. 2024 Nov 14;16:128. doi: 10.1186/s13321-024-00908-y (PMC11566221; doi:10.1186/s13321-024-00908-y)
Supplement: Supplementary file 1 — Additional file 1. [file 13321_2024_908_MOESM1_ESM.pdf]

# QSPRpred: a Flexible Open-Source Quantitative Structure-Property Relationship Modelling Tool – Supporting Information

Helle W. van den Maagdenberg[1†], Martin Šícho[1,2†], David Alencar Araripe[1,3], Sohvi Luukkonen[1,4], Linde Schoenmaker[1], Michiel Jespers[1], Olivier J. M. Béquignon[1, 5], Marina Gorostiola González[1, 6], Remco L. van den Broek[1], Andrius Bernatavicius[1, 7], J. G. Coen van Hasselt[1], Piet. H. van der Graaf[1, 8], Gerard J. P. van Westen[1*]

[1*]Leiden Academic Centre for Drug Research, Leiden University, Einsteinweg 55, Leiden, 2333 CC, The Netherlands.

[2]CZ-OPENSCREEN: National Infrastructure for Chemical Biology, Department of Informatics and Chemistry, Faculty of Chemical Technology, University of Chemistry and Technology Prague, Technická 5, Prague, A-4040, Czech Republic.

[3]Department of Human Genetics, Leiden University Medical Center, Einthovenweg 20, Leiden, 2333ZC, The Netherlands.

[4]ELLIS Unit Linz and LIT AI Lab, Institute for Machine Learning, Johannes Kepler University, Altenberger Straße 69, Linz, 610101, Austria.

[5]Department of Neurosurgery, Brain Tumor Center Amsterdam, Amsterdam University Medical Center, Cancer Center Amsterdam, De Boelelaan 1117, Amsterdam, 1081 HV, The Netherlands.

[6]Oncode Institute, The Netherlands.

[7]Leiden Institute of Advanced Computer Science Leiden University, Niels Bohrweg 1, Leiden, 2333 CA, The Netherlands.

[8]Certara UK, University Road, Canterbury Innovation Centre, Unit 43 Canterbury, Kent, CT2 7FG, UK.

*Corresponding author(s). E-mail(s): gerard@lacdr.leidenuniv.nl;
Contributing authors: h.w.van.den.maagdenberg@lacdr.leidenuniv.nl;
m.sicho@lacdr.leidenuniv.nl; d.figueiredo.vidal@lacdr.leidenuniv.nl;
luukkonen@ml.jku.at; l.schoenmaker@lacdr.leidenuniv.nl;
m.jespers@lacdr.leidenuniv.nl; o.j.m.bequignon@lacdr.leidenuniv.nl;
m.gorostiola.gonzalez@lacdr.leidenuniv.nl;
r.l.van.den.broek@lacdr.leidenuniv.nl;
a.bernatavicius@liacs.leidenuniv.nl; coen.vanhasselt@lacdr.leidenuniv.nl;
p.vandergraaf@lacdr.leidenuniv.nl;
†These authors contributed equally to this work.

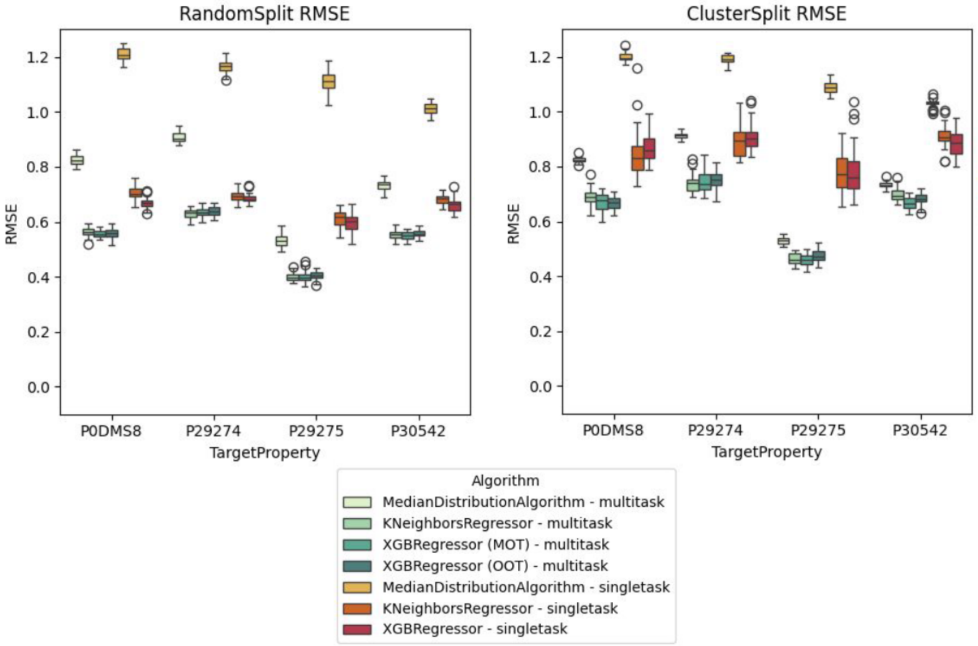


**Fig. 1** Root Mean Squared Error (RMSE) calculated for each replica in different benchmarking runs conducted in Experiment 1.
